# Low Back Pain Assessment Based on Alpha Oscillation Changes in Spontaneous Electroencephalogram (EEG)

**DOI:** 10.1155/2021/8537437

**Published:** 2021-07-01

**Authors:** Li Feng, Hanlei Li, Hongyan Cui, Xiaobo Xie, Shengpu Xu, Yong Hu

**Affiliations:** ^1^Institute of Biomedical Engineering, Chinese Academy of Medical Sciences and Peking Union Medical College, Tianjin 300192, China; ^2^Department of Orthopaedics and Traumatology, Li Ka Shing Faculty of Medicine, University of Hong Kong, Hong Kong, China

## Abstract

Objectively and accurately assessing pain in clinical settings is challenging. Previous studies showed that alpha oscillations of electroencephalogram data are correlated with subjective perceived pain. Based on this finding, this study is aimed at assessing chronic low back pain based on alpha oscillations. Multichannel electroencephalogram data were recorded from 27 subjects with chronic low back pain under the simple conditions of closing eyes or opening eyes. Spectral analyses were conducted to extract the alpha band responses, and the alpha powers were calculated for the two conditions, respectively. Normalized alpha power was calculated by subtracting the alpha power in the eyes-open condition from that in the eyes-closed condition. The correlation between the alpha power and the subjective pain intensity was evaluated in frontal, central, and posterior regions. The normalized alpha power in the central region was negatively correlated with the subjective pain intensity (*R* = −0.50, *P* = 0.01), with the strongest correlation occurring at the Cz electrode (*R* = −0.59, *P* = 0.04). The correlation analysis results demonstrated the possibility of using the differences of alpha spectral power between eyes-closed and eyes-open conditions as a measure for assessing chronic low back pain. The findings suggest that the normalized alpha power in the central region may be used as a measurable and quantitative indicator of chronic pain for clinical applications.

## 1. Introduction

Pain, especially chronic pain, is one of the biggest public health problems in our society [1]. A survey by the National Center for Health Statistics found that low back pain (LBP), migraine or severe headache, and joint pain were the most common types of chronic pain in clinical practice [2]. Thus, an effective technique to measure and quantify LBP is needed to achieve better diagnosis and management in clinical settings [3].

Because pain is a subjective individual experience, self-reported pain intensity is considered the gold standard for evaluating pain in clinical situations [1, 2]. However, the clinical pain measure always needs careful evaluation and a skillful clinician to guide [4]. Besides, self-reports of pain intensity are not available for some vulnerable populations, who have severe cognitive or communicative impairments and cannot provide self-reports [5]. Hence, an objective measure of pain intensity that is expected to be associated with standard measures, such as visual analog scale (VAS) [1], can complement self-reports that would be useful in clinical practice, such as to monitor the effect of an analgesic drug or track the recovery of the nociceptive system in noncommunicative patients [5, 6].

Electroencephalography (EEG) is a noninvasive monitoring technique that is widely used to probe neurological disorders with high temporal resolution. In recent decades, techniques have been developed to objectively quantify subjective perceived pain through cortical measures derived from EEG responses to brief, phase-locked noxious stimuli [7, 8]. Since brain activity in multiple brain regions elicited by transient painful stimuli is often correlated with pain intensity, researchers are motivated to identify brain activity features that could serve as biomarkers for objective pain assessment [9]. However, such brief stimuli may be limited in their ability to reliably simulate natural and clinically painful experiences, even when considering acute pain [8]. Therefore, a growing number of studies have focused on developing an objective approach to measuring chronic pain by using sustained pain stimulation, which may evoke non-phase-locked cortical responses similar to those observed in EEG data associated with chronic pain [10–12].

Recording and characterizing cortical electrophysiological responses to chronic pain require measurements other than event-related potentials, namely, continuous EEG. Continuous EEG data are commonly analyzed by transforming them from the time domain to the frequency domain. EEG at different frequency bands including theta, beta, and gamma has been reported to be related to pain perception [13–16], even though there has not been a clear consensus determining which rhythmic band has the most reliable correlation with different levels of elicited pain. Compared with other frequency domains, alpha band oscillations (8–13 Hz) are the most commonly explored [17] (considering that gamma waves are generated deep in the brain and are therefore not easy to record with scalp EEG [18], while data concerning pain-related beta EEG activity are scarce [16, 19, 20]). Furthermore, resting-state EEG recording of alpha oscillations is found to be stable over time [21], highly heritable [22], and unique to an individual to the extent that it could serve as a “statistical signature” [23]. It has been reported that experimentally induced transient pain elicits a decrease in alpha power [9, 11, 21]. Compared with transient noxious painful experiences, findings regarding the effect of tonic pain on alpha oscillatory activity have been inconsistent: some studies have indicated that alpha rhythm induced by tonic pain is suppressed in frontal-central or parietal-occipital regions [15, 24–26], whereas others have reported that alpha oscillations are enhanced over these cortical regions [14, 27]. Although there is no clear consensus regarding how alpha power changes as sustained stimuli are processed, alpha band oscillations are generally considered to be correlated with different levels of pain [18]. Moreover, it has been found that the frequency corresponding to the maximum alpha power at rest predicts an individual's responsiveness to tonic noxious heat stimuli [24].

This previously reported relationship between alpha oscillations and subjective perceived pain encouraged us to investigate whether alpha oscillations obtained from continuous resting EEG are associated with subjective reports of perceived pain in chronic pain patients. However, our previous studies were limited in that the subjects were healthy adults, so tonic noxious stimuli were used to induce pain and elicit the EEG oscillations [15, 26]. Although experimentally induced tonic pain stimuli better resemble the sensory experience in a clinical setting than brief stimuli, they may not involve the same neurological responses as pain [18, 19, 28]. Some electrophysiological and brain imaging studies on patients suffering from chronic pain reported inconsistent findings of the changes in spontaneous oscillatory activity and their association with pain intensity [13, 29–31]. Most of these preliminary studies were conducted in patients suffering from the pain of different origins, and data analyses were not standardized. Therefore, further investigation is needed to expand the previous findings to the pain population. Accordingly, in the present study, we aimed to investigate whether alpha oscillation, an objective neurophysiological parameter, could serve as a measurable and quantitative indicator associated with chronic LBP.

It is well known that alpha activity in EEG data is dominant in normal individuals in the eyes-closed (EC) resting condition and suppressed in the eyes-open (EO) condition [32]. Numerous prior studies have examined the differences between EEG data obtained in the EC and EO conditions in the resting state to determine appropriate baseline readings for protocol development [33]. In general, the EC condition is recommended as a baseline resting state for EEG measurements in various experimental designs, especially those that do not involve tasks with visual stimulation [32]. In addition, Barry and De Blasio [34] reported that, compared with the other bands, the alpha band exhibits a stable widespread reduction in activity from the EC to the EO condition with no topographic changes, allowing the EC and EO data to be pooled across times for subsequent analyses. These findings may imply that compared with the direct recording of EEG oscillations under the EO condition according to a traditional experimental protocol, employing the EC/EO experimental paradigms in which the EC condition is considered a baseline resting state is promising to obtain a relatively stable normalized alpha power. Therefore, we examine possible EEG oscillations in the alpha band (alpha power under the EO condition or normalized alpha power) that is associated with the subjective perceived chronic LBP. We also compare the performance of different parameters for pain assessment, then propose an appropriate one.

## 2. Materials and Methods

### 2.1. Subjects

Twenty-seven patients with chronic LBP were recruited in this study, including 20 females and 7 males. Their mean age was 44.6 ± 2.3 (mean ± SD) years. None of the patients had a history of neurological or psychiatric disease, without previous history of neurological or spinal surgery. They were diagnosed as LBP without specific neuromusculoskeletal pathology, after a careful clinical assessment by experienced clinicians. All subjects have been suffering LBP with an average duration of 5.6 years (range 2–20 years). Subjects to be included in this study were asked to read the study protocol carefully. Those subjects that cannot understand the protocol to follow the guidance of clinicians very well were excluded.

Each subject provided informed written consent before each experiment. The study was conducted according to the guidelines of the Declaration of Helsinki. The experimental protocol was approved by the Institutional Review Board of the University of Hong Kong and West Cluster of Hospital Authority (UW 08-181). The study was registered on the Clinical Trial Registry (https://register.clinicaltrials.gov/) with the registration number NCT03511404.

### 2.2. EEG Recording

Continuous EEG recording was conducted using a 64-channel Neuroscan System (Compumedics Limited, Victoria, Australia) using an electrode cap according to the 10-20 system. The system was set up with a band-pass filter in the range of 0.01–100 Hz and a sampling rate of 5000 Hz. An electrode placed on the nose was used as the reference channel, and the impedance at each electrode position was kept below 10 k*Ω*. Four surface electrodes were placed to record electrooculographic signals: one pair was placed lateral to the outer corner of the right and left orbit at a distance of 1 cm, and the other pair was placed over the upper and lower eyelids.

### 2.3. Experimental Procedure

Each subject was seated in a comfortable armchair in a quiet and temperature-controlled room. The subject was familiarized with the pain rating scale and the experimental procedures before the experiment, under clear guidance of an experienced physiotherapist. During the experiment, each subject began with the resting EC followed by EO baseline periods of 5 min each. For the EO condition, subjects were instructed to stay relaxed but alert. After the EEG recordings, subjects were asked to verbally rate their perceived pain intensity on a 0–10 VAS, in which 0 was defined as “no pain sensation” and 10 as “the worst imaginable pain”.

### 2.4. EEG Data Analysis

**Figure 1:**
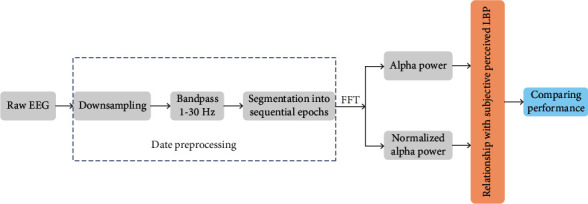
Flowchart describing the procedure used for EEG data analysis.

The data analysis procedure in the present study is illustrated in Figure 1. Raw EEG data were first preprocessed by downsampling, band-pass filtering, and segmenting into sequential epochs. Then, spectral analysis was employed to obtain the spectral powers in the alpha band (both alpha power under the EO condition and normalized alpha power). Finally, the performance of different parameters for LBP assessment was compared by associating with subjective perceived LBP.

#### 2.4.1. Data Preprocessing

Raw EEG data were processed in the MATLAB environment (MathWorks Inc., Massachusetts, United States). After downsampling the data to 1000 Hz, the data for each condition (EC and EO) were band-pass filtered in the range of 1–30 Hz. Then, each 5 min EEG recording was segmented into 300 sequential 1 s epochs, of which the first five were rejected to avoid evoked responses associated with the action of opening or closing the eyes. Each epoch was baselined across its duration and rejected if the activity at any scalp site exceeded ±100 *μ*V at any time. EEG segments contaminated with strong muscle artifacts were manually eliminated by visual inspection. Epochs contaminated by eye blinks and movements were corrected using independent component analysis (ICA) in EEGLAB V13.0, an open-source toolbox running in the MATLAB environment. The main criteria to determine whether a component is artifact are the scalp map, the component time course, and the component activity power spectra [35]. In all datasets, independent components with a large electrooculogram (EOG) channel contribution and a frontal scalp distribution were removed. After preprocessing and selection, the remaining artifact-free, 1 s epochs from all 27 subjects were selected for further analyses.

#### 2.4.2. Spectra Extraction

As the present study is aimed at investigating the relationship between alpha band activities and subjective perceived LBP, only data concerning the alpha frequency range (8-13 Hz) are presented. The segmented EEG epochs of each subject in each condition were transformed from the time domain to the frequency domain, using fast Fourier transformation (FFT), yielding power spectra (in *μ*V^2^). For each subject and electrode, the obtained power spectra over the frequency band were summed to summarize the spectral power values of the alpha band for each epoch; this procedure was repeated for all conditions. For each electrode, the obtained single-epoch power spectra were averaged across epochs to enhance the signal-to-noise ratio. Then, for each subject and condition, the power spectra were averaged in three regions: frontal (F3, Fz, F4, FC1, and FC2), central (C3, C1, Cz, C2, C4, CP1, CPz, and CP2), and posterior (P3, Pz, P4, O1, Oz, and O2). These electrodes were selected because of ample prior evidence indicating their substantial relevance in experimentally induced tonic pain [10, 24, 25, 27, 36].

Two calculations for the alpha power were performed to investigate various possible cortical parameters potentially associated with subjective perceived chronic LBP. The averaged alpha power value for each subject was obtained for the EC and EO conditions separately using the spectral analysis. The obtained values from the EO condition were utilized as one variable for correlation analysis to evaluate the relationship with the VAS scores. The second parameter investigated was the differences between the alpha band powers in the EC and EO conditions derived by alpha power normalization (here, the differences between the absolute alpha power values in the EC and EO conditions are the normalized alpha powers for statistical analysis).

#### 2.4.3. Statistical Analysis

All statistical analyses were conducted in the MATLAB environment. To examine the global changes in alpha activity, the obtained power differences from each subject were group-averaged in the three main regions (frontal, central, and posterior). The mean and standard deviation (SD) were computed for each parameter.

The relationships between EEG responses and subjective pain intensities (VAS scores) were assessed by linear regression and Pearson correlation analyses, and the *P* values were corrected for multiple comparisons using the Bonferroni method. Specifically, two correlations were conducted to determine (1) whether the alpha power values obtained from resting EEG data are associated with subjective perceived chronic LBP and (2) which parameter (alpha power from the EO condition or normalized alpha power) is more strongly correlated with pain intensity. Thus, the first relationship to be assessed was between the alpha power values from the EO condition and VAS scores, and the second relationship to be assessed was between the absolute alpha power reduction from the EC to the EO condition and the VAS scores. Statistical significance was set at *P* < 0.05.

## 3. Results

### 3.1. Spectral Power in Different Conditions

**Figure 2:**
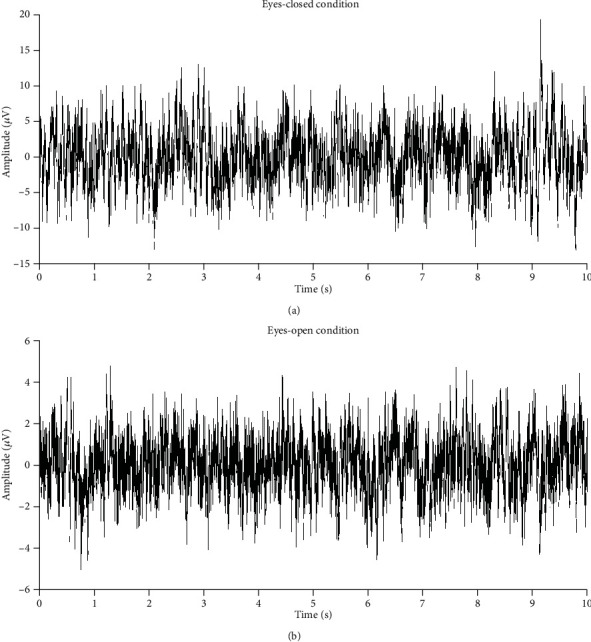
Representative 10 s long preprocessed EEG recordings derived from the Cz electrode in one randomly chosen subject under (a) eyes-closed (EC) and (b) eyes-open (EO) conditions.

**Figure 3:**
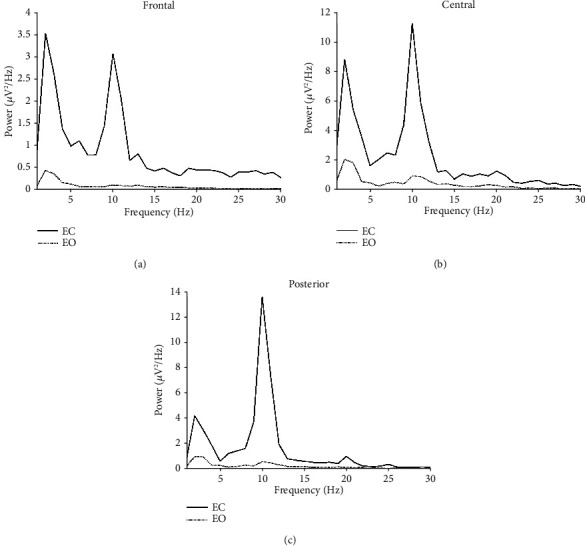
Group-averaged EEG power spectral densities under eyes-closed (EC) and eyes-open (EO) conditions in (a) frontal, (b) central, and (c) posterior regions.

Figure 2 shows 10 s long preprocessed EEG signals derived from the Cz electrode in one randomly chosen subject under the EC and EO conditions.  Figure 3 shows the mean alpha power spectral densities under the EC and EO conditions averaged over the frontal (F3, Fz, F4, FC1, and FC2), central (C3, C1, Cz, C2, C4, CP1, CPz, and CP2), and posterior (P3, Pz, P4, O1, Oz, and O2) electrodes. The spectral power for the EO condition was significantly decreased compared with those of the EC condition within the alpha band in the three main regions.

### 3.2. Global Alpha Power Change between Conditions

**Figure 4:**
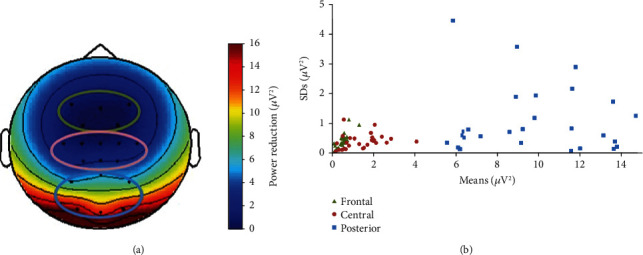
(a) Global power differences between eyes-closed (EC) and eyes-open (EO) conditions in the alpha frequency band and statistical results in the three main regions. (b) The means and SDs of the power differences for each subject in the three regions.

To examine the global changes in alpha power, the absolute power differences in the alpha band at each electrode were calculated and then group-averaged over the frontal, central, and posterior regions. The topographic effects of the summarized spectral power reduction within the alpha band (8–13 Hz) are shown in Figure 4(a). The results show a global suppression in the alpha rhythms between the EC and EO conditions, especially in the occipital cortices. There was a local increase in the frontal area, but a decrease when computed over all electrodes. The means and SDs of the power differences for each subject in the three regions are shown in Figure 4(b). The group-averaged summarized alpha spectral power reduction was 0.36 ± 0.20 *μ*V^2^ in the frontal region, 1.52 ± 0.65 *μ*V^2^ in the central region, and 9.81 ± 3.41 *μ*V^2^ in the posterior region.

### 3.3. Correlation between Alpha Power and Subjective Pain Rating

**Figure 5:**
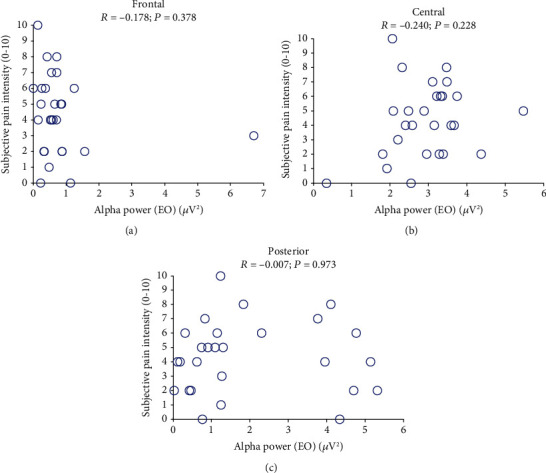
Relationships between alpha power under eyes-open (EO) condition and subjective intensity of pain perception in (a) frontal (*R* = −0.178, *P* = 0.378), (b) central (*R* = −0.240, *P* = 0.228), and (c) posterior (*R* = −0.007, *P* = 0.973) regions.

**Figure 6:**
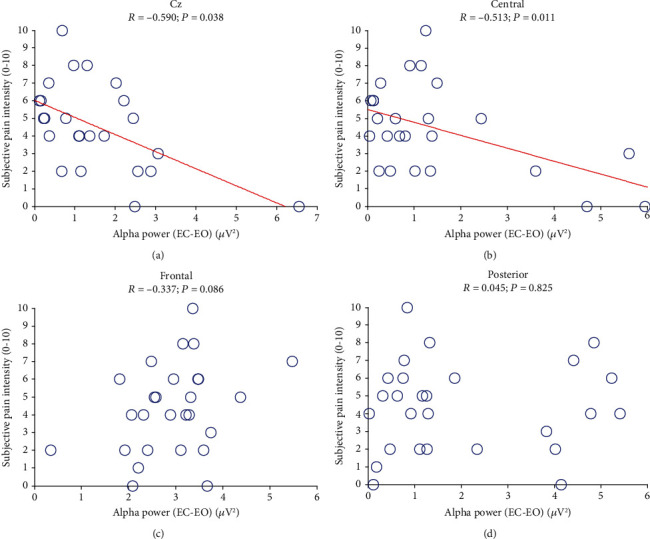
Relationships between alpha power reduction from eyes-closed (EC) condition to eyes-open (EO) condition and subjective intensity of pain perception in (a) central (*R* = −0.513, *P* = 0.011), (b) Cz electrode (*R* = −0.590, *P* = 0.038), (c) frontal (*R* = −0.337, *P* = 0.086), and (d) posterior (*R* = 0.045, *P* = 0.825) regions.

The correlations between the alpha spectral powers of each subject obtained in the EO condition and the subjective pain intensity are shown in Figure 5; no linear relationship was observed in any region (*P* > 0.05). The relationships between the power differences between EC and EO conditions and VAS scores are displayed in Figure 6. The reduction in the alpha power in the central region (C3, C1, Cz, C2, C4, CP1, CPz, and CP2) was negatively correlated with the subjective pain rating (*R* = −0.513, *P* = 0.011; Figure 6(a)), and the strongest correlation was observed with the signal from the Cz electrode (*R* = −0.590, *P* = 0.038; Figure 6(b)). However, no such linear relationship was observed between the VAS scores and the alpha powers in the frontal (*R* = −0.337, *P* = 0.086; Figure 6(c)) or posterior electrodes (*R* = 0.045, *P* = 0.825; Figure 6(d)).

## 4. Discussion

In this study, we evaluate the relationship between the alpha power, an objective neurophysiological parameter, and subjective reports of experienced pain in people with chronic LBP. The observed decrease in alpha power in the central region under the conditions of closing and opening eyes in a resting state had a negative linear relationship with the subjective pain rating. Furthermore, the correlation between the normalized alpha power and VAS scores indicated that the alpha band spectral change between the EC and EO conditions from the resting EEG data might serve as a measurable and quantitative indicator for subjective perceived chronic pain.

For feature extraction within the alpha frequency band, we focus on the alpha spectral power in particular. Compared with other frequency domains, alpha band oscillations (8–13 Hz) are the most commonly explored, and the decrease in this metric has been repeatedly associated with the administration of noxious stimuli [15, 17, 22, 24, 27, 37]. It is noted that gamma oscillation features also have been reported to be related to pain perception and considered a potential pain assessment tool [14, 15, 18]. However, the signal-to-noise ratio of gamma oscillation is poor [27], since higher frequency data are normally and easily contaminated by a lot of nonneural artifacts (e.g., cranial and ocular muscle activity) with practical difficulty in clinical use, whereas alpha activity is related to the amount of experimentally induced pain in the somatosensory cortex, more reliably measured by scalp [18]. Based on the previous findings [32–34], to identify stable and reliable alpha band activities, the EC and EO conditions were evaluated experimentally. Using the simple modulation of closing and opening the eyes, the alpha power was directly obtained for each condition, and the power changes in the alpha band were obtained by computing the absolute power reductions between the conditions. The obtained power differences from each subject were group-averaged in the frontal, central, and posterior regions to evaluate global changes in the alpha activity. Statistical analyses of the means and SDs of the power changes showed a global suppression in the alpha rhythms from the EC to the EO condition. As observed in a study of healthy people [34], the alpha reduction in patients with chronic LBP was more considerable in the posterior region, which suggests arousal in the EO condition compared with the EC condition. As described by Berger in the 1920s [38], the alpha rhythmic activity is the strongest electrophysiological signal measured from the surface of the awake human brain. High levels of alpha activity were previously interpreted as cortical idling because alpha activity increases in brain areas that are not engaged in a task.

To investigate whether alpha power is associated with subjective perceived chronic LBP and propose a more useful parameter, the relationships of the VAS scores with two different variables (alpha power in the EO condition and normalized alpha power) were investigated. The results of the correlation analysis showed that increased subjective perceived chronic LBP was solely associated with decreased oscillations of the alpha power between the EC and EO conditions (resting state) in the central region. Consistent with our findings, reductions in the alpha band were observed in previous works on tonic experimental pain using both heat- and cold-presser tests [10, 15, 27, 36, 39–42]. Compared with previous findings based on stimuli-evoked pain responses, the results of our study demonstrate that the suppression of alpha oscillations extracted from spontaneous EEG signals has a linear relationship with the subjective pain intensity in chronic LBP patients. This finding may indicate that alpha power differences between EC and EO conditions recorded at rest could reveal individual predispositions for pain responsiveness. Unlike previous studies [14, 15, 24, 27, 36, 39], in which EPs were induced by long-lasting stimuli in healthy subjects, we collected spontaneous resting EEG data from chronic LBP patients. Because this procedure does not require a task, the EEG data can be obtained much more readily from patients without training on performing specific tasks. Thus, the approach used here may serve as a simple and useful clinical methodology for measuring subjective pain intensity. In addition, an ongoing debate regarding nociception-associated changes in cortical oscillatory activity is whether the phenomena observed are elicited by pain or merely the saliency of external stimuli. Hu et al. [37] have shown that the alpha power fluctuations induced by phasic stimulation can reflect both sensory-related and task-related cortical processes, where sensory-related alpha event-related desynchronization (*α*-ERD) is predominantly located over contralateral central electrodes, and task-related *α*-ERD is located at posterior parietal and occipital electrodes. However, it remains unclear how tasks may affect the pain-induced changes in spontaneous oscillatory activity. Because the participants in our experiment were not asked to perform a task and did not receive any external stimuli, we can rule out the saliency effect.

Our results are also in accordance with EEG-based measurements performed in chronic pain patients, which found decreased cortical inhibition in pain-controlling regions [8]. However, some studies in patients with fibromyalgia, a disease in which mechanisms generating chronic pain are still elusive, reported increased or unchanged cortical inhibition [31, 43]. Other studies reported rather increased beta oscillations [16] and lower gamma oscillations [14] in neurogenic pain. Most of these studies were conducted using varied electrophysiological techniques and brain imaging methods, and the analysis frequency domain was segmented with different cutoffs. In addition, data acquired with eyes-closed condition were pooled together with those recorded in eyes-open condition, which is reported to dramatically modify both the topography and the magnitude of different frequency components [33]. In our study, selected data was recorded only in alpha frequency segmentation and normalized using validated methods [32–34]. The results imply that the proposed novel experimental setting used in the present study enables the effective extraction of more stable and reliable alpha oscillations than the traditional approach, which allows for meaningful correlations to be identified. Furthermore, this neurophysiological-psychophysical relationship broadens the existing knowledge regarding prolonged pain quantification based on alpha oscillations by delineating a distinct functional role of their power in representing subjective experiences of tonic pain in addition to their previously explored characteristics [37, 39, 44]. The identification of electrophysiological signatures encoding how the cortex processes the experience of chronic pain could indeed open a window to study the cortical process underlying the pain function in humans [45]. In clinical practice, this understanding also would make it possible to predict subjective pain intensity objectively and help explore the pathological mechanisms of chronic pain and achieve pain relief by modulating the oscillatory activities using neurofeedback techniques, with the investigation of cortical oscillatory activities on chronic pain patients [18].

The alpha power in the central region was the most responsive to changes in the subjective pain intensity. While it may be difficult to associate electrical activities recorded from the scalp with specific brain sources, their distribution is consistent with pain-related activities in the somatosensory association areas located in the parietal operculum and insula. Similar findings have been reported in the study of identifying oscillatory activities from intracerebral EEG by using tonic thermonociceptive stimulation [27, 36, 42]. Previous studies have characterized the cortical representation of pain in the primary sensorimotor areas (S1/M1) using intracortical evoked potentials from epileptic patients [46]. Our finding suggests that the central processing of chronic pain within an individual can be investigated through the neural function of their pain network at rest, enabling the prediction of subjective pain responsiveness without using external stimuli. This result also complements the report of alpha band amplitude study at the same location in patients with chronic pain [17]. Based on the close association between alpha oscillatory activity and cortical excitability [37, 44], the observed significant suppression in the central region (especially at the Cz electrode) may indicate elevated excitability in the sensorimotor cortex. Additionally, the general role of alpha rhythms in inhibiting different processes within the brain can be used as a framework to interpret the results [47, 48]. Suppressing alpha band power is thought to elicit a top-down feedback effect in the connectivity of different cortical regions, which allows for the information to flow from the sensory area to other relevant functional areas in response to pain [49, 50]. Moreover, the finding revealed an increase in central processing in the case of chronic LBP, based on the close association between alpha oscillatory activity and cortical excitability [37, 44, 51].

However, even though our study has been carefully designed with a good clinical education and guidance for pain assessment to minimize individual differences, the individual variance such as personality or trait differences may cause subjective differences in pain perception [27]. The absence of a higher statistical power in the correlation results might be due to the effect of the outliers, considering the limited number of our trials. Additionally, in terms of our subjects' gender, we had a greater percentage of female participants. In the present study, we did not take gender balance as an influencing factor considering that the previous study did not observe any significant fixed effect from gender differences in EEG features of pain [22]. More balanced recruitment is expected to be conducted in the future to test gender effects.

## 5. Conclusions

In this study, we found that suppression of the alpha frequency band in the central electrodes under the conditions of closing and opening eyes in a resting state had a negative linear relationship with subjective perceived pain in chronic LBP patients. The degree of suppression may reflect the level of subjective pain intensity. These preliminary results, although needing confirmation, give the possibility for the objective and straightforward assessment of chronic pain by means of a short EEG recording at rest.

There were several limitations of the present work that could be addressed in future studies. First, as subjects were chronic low back pain patients, further investigation is needed to expand the current findings to other clinical pain populations. To increase the power of the approach and be able to detect reliable changes in alpha oscillations, more trials might be necessary. In addition, the mechanisms of underlying neural activity in chronic pain still need to be clarified. Extensive EEG source localization and connectivity analysis could be implemented to further explore these processes. Finally, although the present study focused on alpha band frequency due to their association with pain, additional bands should be considered to identify relationships between signals at other frequencies with subjective pain perception.

## Data Availability

The datasets generated or analyzed during the current study are not publicly available due to the terms of consent to which the participants agreed but are available from the corresponding author on reasonable request.
